# Is the rotation of the femural head a potential initiation for cutting out? A theoretical and experimental approach

**DOI:** 10.1186/1471-2474-12-79

**Published:** 2011-04-22

**Authors:** Andreas Lenich, Samuel Bachmeier, Lukas Prantl, Michael Nerlich, Jochen Hammer, Edgar Mayr, Amir Andreas Al-Munajjed, Bernd Füchtmeier

**Affiliations:** 1 Department of Trauma Surgery, Klinikum Augsburg, Augsburg 86156, Germany; 2 Biomechanic Research Regensburg, University of Applied Sciences Regensburg, Regensburg 93053, Germany; 3 Department of Trauma Surgery, University of Regensburg, Regensburg 93053, Germany; 4 Department of Trauma Surgery, Klinikum Rechts der Isar, Technical University of Munich, Munich 81675, Germany

**Keywords:** proximal femur fractures, osteosynthesis, center-center-position, cutting out

## Abstract

**Background:**

Since cut-out still remains one of the major clinical challenges in the field of osteoporotic proximal femur fractures, remarkable developments have been made in improving treatment concepts. However, the mechanics of these complications have not been fully understood.

We hypothesize using the experimental data and a theoretical model that a previous rotation of the femoral head due to de-central implant positioning can initiate a cut-out.

**Methods:**

In this investigation we analysed our experimental data using two common screws (DHS/Gamma 3) and helical blades (PFN A/TFN) for the fixation of femur fractures in a simple theoretical model applying typical gait pattern on de-central positioned implants. In previous tests during a forced implant rotation by a biomechanical testing machine in a human femoral head the two screws showed failure symptoms (2-6Nm) at the same magnitude as torques acting in the hip during daily activities with de-central implant positioning, while the helical blades showed a better stability (10-20Nm).

To calculate the torque of the head around the implant only the force and the leverarm is needed (N [Nm] = F [N] * × [m]). The force F is a product of the mass M [kg] multiplied by the acceleration g [m/s^2^]. The leverarm is the distance between the center of the head of femur and the implant center on a horizontal line.

**Results:**

Using 50% of 75 kg body weight a torque of 0.37Nm for the 1 mm decentralized position and 1.1Nm for the 3 mm decentralized position of the implant was calculated. At 250% BW, appropriate to a normal step, torques of 1.8Nm (1 mm) and 5.5Nm (3 mm) have been calculated.

Comparing of the experimental and theoretical results shows that both screws fail in the same magnitude as torques occur in a more than 3 mm de-central positioned implant.

**Conclusion:**

We conclude the center-center position in the head of femur of any kind of lag screw or blade is to be achieved to minimize rotation of the femoral head and to prevent further mechanical complications.

## Background

Although the variety of devices for unstable trochanteric and subtrochanteric femoral fractures is rising annually and improvements in surgical techniques and implant modifications have been performed, serious clinical and mechanical complication rates up to 20% are still found in the fixation of proximal femur fractures [1-4]. Clinical complications include the rotation of the femoral head and the cut-out phenomenon of the fracture fixation bolt (cutting out rate 3-18%). Previously we investigated the fixation of several proximal femur osteosyntheses using clinical and experimental studies indicating that a helical blade shows a better fixation of proximal femur fractures [5-7]. A recent study also concluded that a helical blade leads to a superior anchorage with a reduction in cut-out complications [8].

Since cut-out still remains one of the major clinical challenges in the field of osteoporotic proximal femur fractures [1,9], remarkable efforts are made in developing superior treatment concepts. However, the mechanics how these complications have not been fully understood in detail. There are a lot of investigations published on fixation stability of different implant types about axial load but only few about rotational stability.

In this investigation we compared our previously published experimental data in a simple theoretical model using a de-central implant position during typical gait pattern. This simple model allowes to calculates the high torque rate in decentral implant position by using the weight of a patient and the distance between the implant and the center of the head of femur. This torque figure might explane the mechanism of the cutting out by comparing it with the experimental data about the rotational resistance of the different implants.

We hypothesize that a decentral implantation of the lag screw can lead to rotation of the femoral head around the implant. This mechanism can initiates a reaction leading to severe complication like a cut-out of the implant.

## Methods

The experimental approach to analyze various implants for proximal femur fractures was previously described [5,6] only the results were used and the biomechanical tests are not part of this study. Briefly, human cadaver femoral heads (n = 24) were used. The specimen were fixed in a special conic embedding system with cement. All used lag screws of different proximal femur osteosynthesis (Proximal Femur Nail Antirotation (PFN A/Synthes/Umkirch, Swiss), Trochanter Fixation Nail (TFN/Synthes/Umkirch, Swiss), Gamma 3 Nail (Stryker/Freiburg, Germany), Dynamic Hip Screw (DHS/Synthes/Umkirch, Swiss)) were implanted into the femoral heads according to the manufacturers manuals using the original instruments. A forced axial rotation of the femoral head was executed with a speed of 1°/sec. During the rotation the axial movement of the embedded sample was blocked. This experiment was divided in left (+60°) and right (-60°) rotations. All tests were performed in a biaxial servo hydraulic test system by Instron with an Interlaken controller, an Instron load and torque cell. Prior to human cadaveric tests the experimental system was calibrated using Sawbone foam blocks (40 × 30 × 30 mm) with a density of 0.20 g/cm3 [5,6].

### Theoretical model

**Figure 1:**
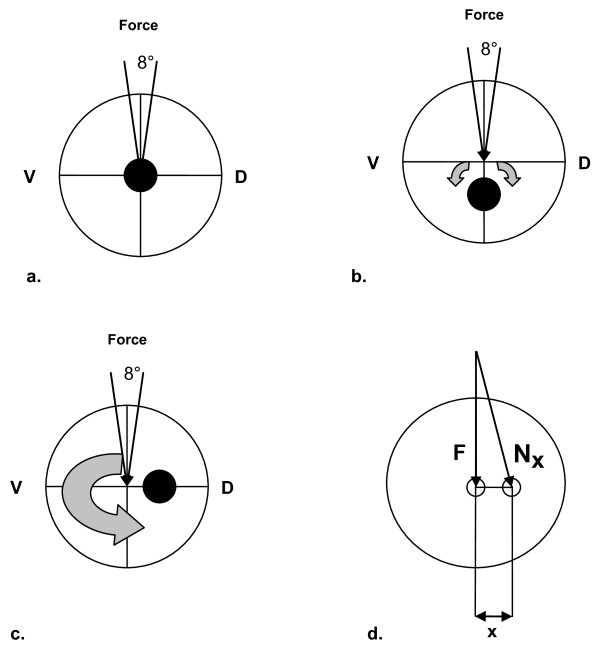
**Schematic 2D representation of the femoral head showing an a. central, b. inferior, c. posterior positioning of the load carrier and d. the example the occurrence of torque Nx with the Force F and the lever x**.

In physics, torque is a vector that describes a force to rotate a corpus about a central point, figure 1. The magnitude of a torque N [Nm] is defined as force F [N] times the length of the lever arm x [m], equation 1. The force F is a product of the mass M [kg] multiplied by the acceleration g [m/s^2^], equation 2.(1)(2)

A theoretical body weight (BW) of 75 kg was used in this model. Loads of 50% (standing) and 250% (normal walking) of the bodyweight (BW) resulting in almost 370N for 50% BW and 1840N for 250% BW have been used simulating daily activities. Using implant position 1 and 3 mm out of the theoretical perfect femur centre torques acting in the proximal femur were calculated.

## Results

**Figure 2:**
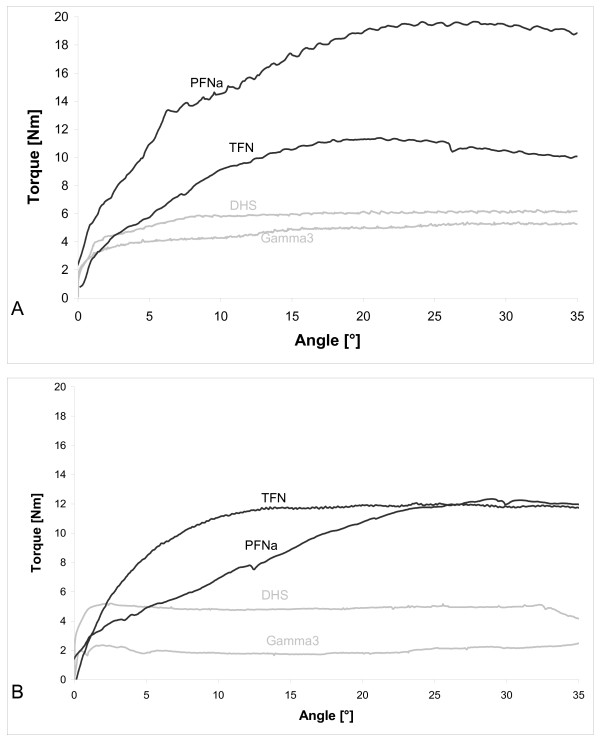
**A) Clockwise rotation (-) and B) Anticlockwise rotation (+) of the human femoral heads with fixed blades (PFNa/TFN) or screw (DHS/Gamma 3) **[5,6]

Figure 2 shows the torque for the femur nails using the A) clockwise and B) anticlockwise rotation. The helical blades (PFN A/TFN) show for both directions higher maximal torques compared to the screws (DHS/Gamma3). Using the clockwise rotation the PFN A shows a higher value of almost 20Nm compared to the TFN with 10Nm, while in the anticlockwise rotation both have identical maxima of 12Nm. Helical blades show a steady increase to the maximum torque, while the screw systems indicate a constant torque level. The screws (DHS/Gamma3) show for the clockwise rotation identical values of around 6Nm, while the DHS (5Nm) has a higher maximal torque anticlockwise then the Gamma3 (2Nm).

**Figure 3:**
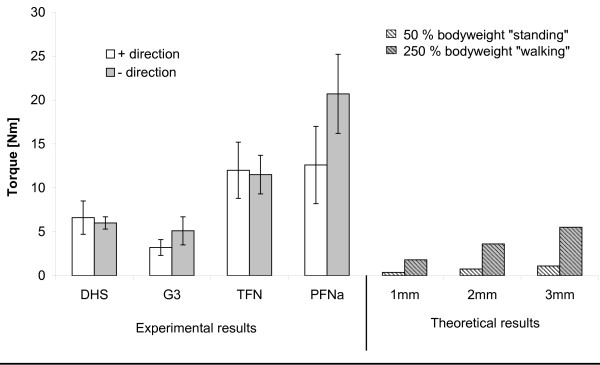
**Summary of all rotation experiments for various implants, Dynamic hip screw (DHS), Gamma 3 (G3), Trochanteric Femur Nail (TFN) and Proximal Femur Nail A (PFN A) in human femoral heads as previously published **[5,6]**compared to theoretical torques acting in the hip during daily activities as standing and walking according to [1o] **.

Figure 3 shows the maximal torques from all experimentally tested femur nails and compares them with calculated values. Using a body weight of 75 kg at 50% BW a torque of 0.37Nm for the 1 mm decentralized position and 1.1Nm for the 3 mm decentralized position of the implant was calculated. At 250% BW torques of 1.8Nm (1 mm) and 5.5Nm (3 mm) have been calculated.

## Discussion

Several new developments have been made to improve proximal femur nails, however, clinical complications as the rotation of the femoral head and in particular the cut-out phenomena are still found [1,8].

In this study we compared our previously published experimental data of two fixation screws (DHS/Synthes^®^, Gamma 3 nail/Stryker^®^) and helical blades (TFN/Synthes^®^, PFN A/Synthes^®^) and compared them to a theoretical model using loads acting in the hip during daily activities [5,6]. The stability of the two screws measured by a forced implant rotation showed torque values in the same magnitude as torques acting in the hip during daily activities with de-central implant positioning, while the helical blades showed a better stability.

Torsion does not occur in the intact center of the femoral head [2]. However, when inducing a fracture in the proximal femur and by fixing this defect with an implant, torsion can occur when this load-bearing implant is not placed in the theoretical center of the femoral head. It has been reported that numerous complications, occur due to poor implant positioning [3]. Human anatomy and variance in human femoral heads however, make an implantation into the theoretical centre almost impossible. Implantation devices improved remarkable in the last years, new developments were focused on the minimal invasiveness though. Additional, rotational instability of intertrochanteric fractures increases with the complexity of the fracture and rotation of the femoral neck fragments leads to an increased complication rate [4].

In the human hip, loads of 50% to 350% of the bodyweight during daily activity have been investigated [10]. In particular cases loads of 900% of the actual bodyweight are expected though. During daily activities 50% (standing) and 250% (normal walking) of the bodyweight (BW) have been seen [10]. We therefore used these values as reference for our experimental data.

Comparing experimental and theoretical results shows that both screw systems fail in the same magnitude as torques occur in a greater than 3 mm de-central positioned implant. As described above a de-central implant position is seen often after surgery. This would lead to many clinical complications with rotated femoral heads. These clinical complications are however, not seen as the majority of complications. Cut-out complications are rather seen in follow-up checks in clinics. We therefore hypothesize that a rotation of the femoral head around the implant initiates a reaction leading to a cut-out of the implant. It is published that in terms of a domino effect, the stability of cancellous bone falls rapidly after the first trabeculars are fractured [11].

A cut out is often seen in clinics and need immediate reaction with revision surgery to prevent further damage in the hip. However, the mechanics and the reason that leads to the cut-out are barely investigated yet. Follow-up studies are still required to investigate the mechanisms that lead to complications with proximal femur osteosynthesis regarding misplacement in mediolateral, anteroposterior and craniocaudal direction [8]. Brunner et al for example descriped in newer implants using helical blades the major mode of failure has been cutting-in through the medial femoral head without loss of reduction [12].

The cut-out of the implant is often seen as a cyclic sintering, creeping or migrating through the first trabecular, then cortical bone. However, assuming a rotation of the femoral head changes all physiological loading conditions within the hip and even single movements may effect a cut-out.

Recently investigations focus also on the implant positioning in the femoral head. The recommendations given in publications and in the documentation provided by implant producers vary with regard to the position of load carriers in the femoral head. In 1959 Cleveland et al. discussed the positioning of osteosyntheses for proximal femur fractures with the conclusion that multiple positions are acceptable and no real difference in the results could be seen [13]. More recently in 1995 Baumgaertner et al. introduced the tip-apex distance theory discussing the implant positioning [14]. Due to varying statements also several manufacturers started to give different instructions in their manuals. Our previous clinical investigations have revealed that the position of the implant prior to fracture reduction plays a decisive role with regard to the complication rate [6].

**Figure 4:**
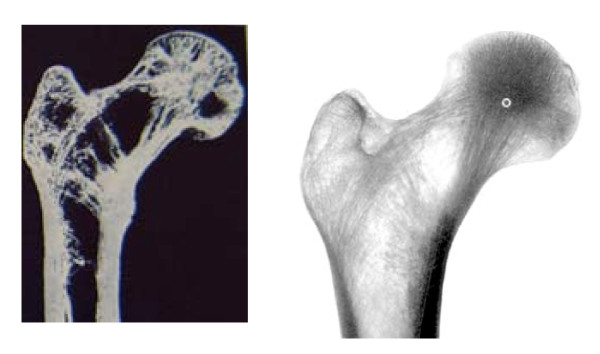
Anatomical bone slice of the proximal femur (85J old male) and x-ray in a.p. projection of the proximal femur

Even mechanical complications after using implants with two proximal compounds like the PFN (Proximal Femur Nail) has been descriped with rotation and cutting out. In our own cases severe osteoporosis has been observed. Osteoporosis means a reduction of cancelous bone fomation and cortex with an enlargement of the bone diameter. One can observe by osteoporosis in the proximal femur region a reduction of the trabecular structure with a concentration of cancelous bone in the center of the head of femur (figure 4). In an osteoporotic head of femur the antirotation screw is placed in an area with less bone structure. This means the best antirotational effect can be achieved by a position of the antirotation screw close to the upper cortex of the oval shaped neck of femur. In our opinion and experience the lag screw should be placed in the center-center position of the head of femur to reduce torque moments and prevent rotation.

This investigation has several limitations. We used a very simplified model without involving surrounding soft-tissue and other factors that might stabilize the fracture. Additional only static loads have been included, while dynamic loads might reduce the life cycles and maximum loads before failure. We see this investigation as a stimulus for further experiments that might clarify the causes for cut-outs as clinical complications.

## Conclusion

We conclude due to de-central implant positioning the load torque can outrun resistance of the cancellous bone around the implant in normal daily activities. The rotation of the head might lead to a cutting out. The biomechanical tests showed the resistance of the bone implant interface depends on the implant design. This study has indicated that the experimental data combined with the theoretical model can illustrate a possible cut out mechanism. We conclude the center-center position in the head of femur of any kind of lag screw or blade is to be achieved to minimize rotation of the femoral head and to prevent further mechanical complications.

## Authors' contributions

The clinical investigations were done by EL, BF and LP under the supervision of EM and MN. The experiments were part of a biomechanical test series done by EL, SB and AA in the biomechanical laboratories of JH, he participated in the design and coordination. All authors read and approved the final manuscript.

## Pre-publication history

The pre-publication history for this paper can be accessed here:

http://www.biomedcentral.com/1471-2474/12/79/prepub
